# A Rare Occurrence of Primary Hepatic Leiomyosarcoma Associated with Epstein Barr Virus Infection in an AIDs Patient

**DOI:** 10.1155/2013/691862

**Published:** 2013-08-19

**Authors:** Haritha Chelimilla, Kanthi Badipatla, Ariyo Ihimoyan, Masooma Niazi

**Affiliations:** ^1^Division of Gastroenterology, Department of Medicine, Bronx Lebanon Hospital Center, Albert Einstein College of Medicine, Bronx, NY 10457, USA; ^2^Division of Pathology, Department of Medicine, Bronx Lebanon Hospital Center, Albert Einstein College of Medicine, Bronx, NY 10457, USA

## Abstract

Primary hepatic leiomyosarcoma is exceedingly rare accounting for less than 1% of the hepatic tumors. Close to 45 cases have been reported in the English literature. Presentation is usually nonspecific and diagnosis is often delayed until tumors reach a large size. This leads to a dismal prognosis. The tumors are not yet fully understood, hence the standard of care is not well defined. Curative resection remains the mainstay of management. Close association of Epstein Barr virus (EBV) induced soft tissue sarcomas is proven, especially in the presence of immunosuppression encountered in HIV/AIDS patients and in posttransplant patients. We herein present a case report of a 54-year-old man diagnosed to have HIV/AIDS and EBV infection admitted to our hospital with complaints of intractable hiccups for more than a week. Extensive workup revealed primary leiomyosarcoma of the liver.

## 1. Introduction

Sarcomas are rare mesenchymal tumors and account for 1% of all adult tumors, of which 5–10% are leiomyosarcomas. Hepatic leiomyosarcomas are unusual tumors. Most of them are metastatic leiomyosarcomas. Primary hepatic leiomyosarcoma is a very rare entity. Primary leiomyosarcomas are known to occur in uterus, retroperitoneum, genital organs, major blood vessels of the body, lungs, and liver [1]. They usually present with nonspecific symptoms and the diagnosis is often delayed resulting in poor prognosis. We herein present a case of a 54- year- old man with HIV/AIDS and EBV infection presenting with intractable hiccups and subsequent workup led to the diagnosis of primary hepatic leiomyosarcoma.

## 2. Case Report

**Figure 1:**
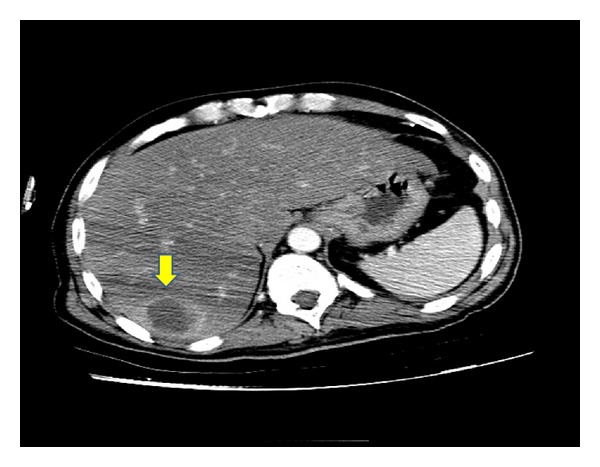
Computer tomography scan with contrast medium showing the liver mass with in the Segment VII of the right lobe with peripheral rim enhancement with central hypodensity.

**Figure 2:**
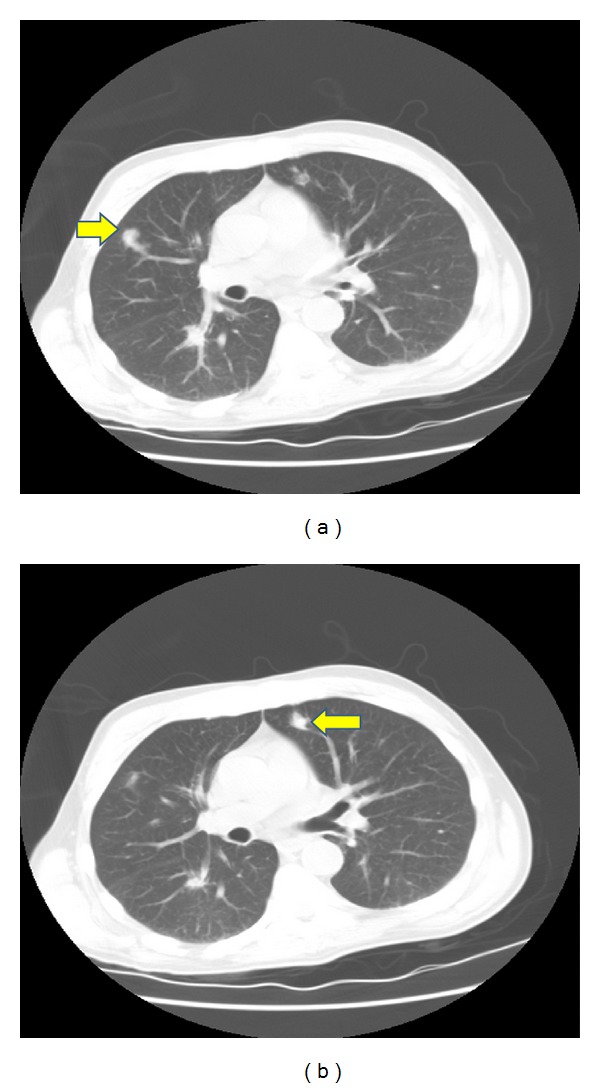
CT chest showing the pulmonary nodules.

**Figure 3:**
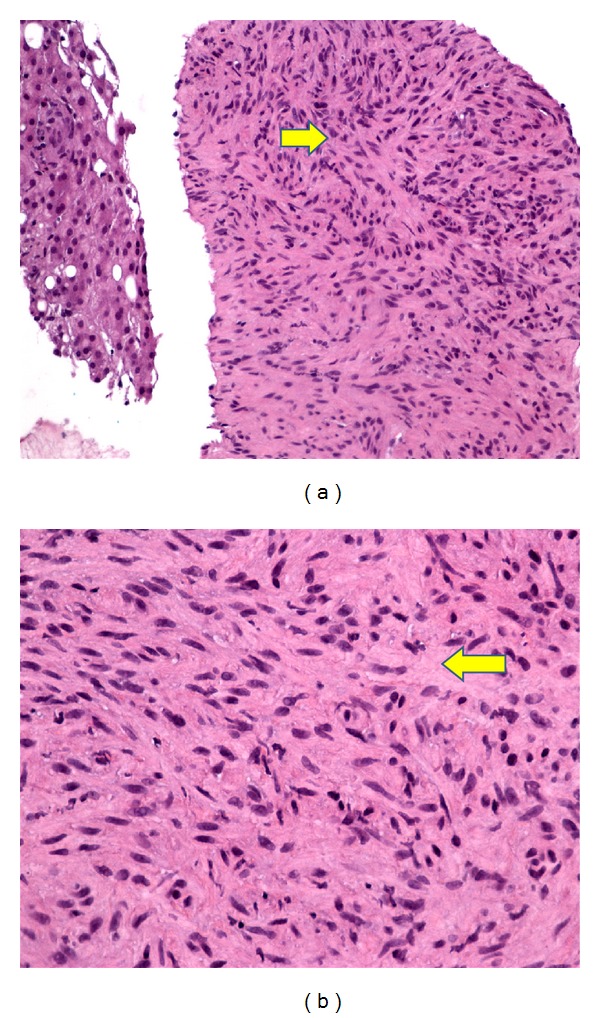
Histopathology of the hepatic mass. (a) Spindle shaped cells distributed in a collagenous stroma. (b) Spindle cells proliferation in interlacing pattern.

**Figure 4:**
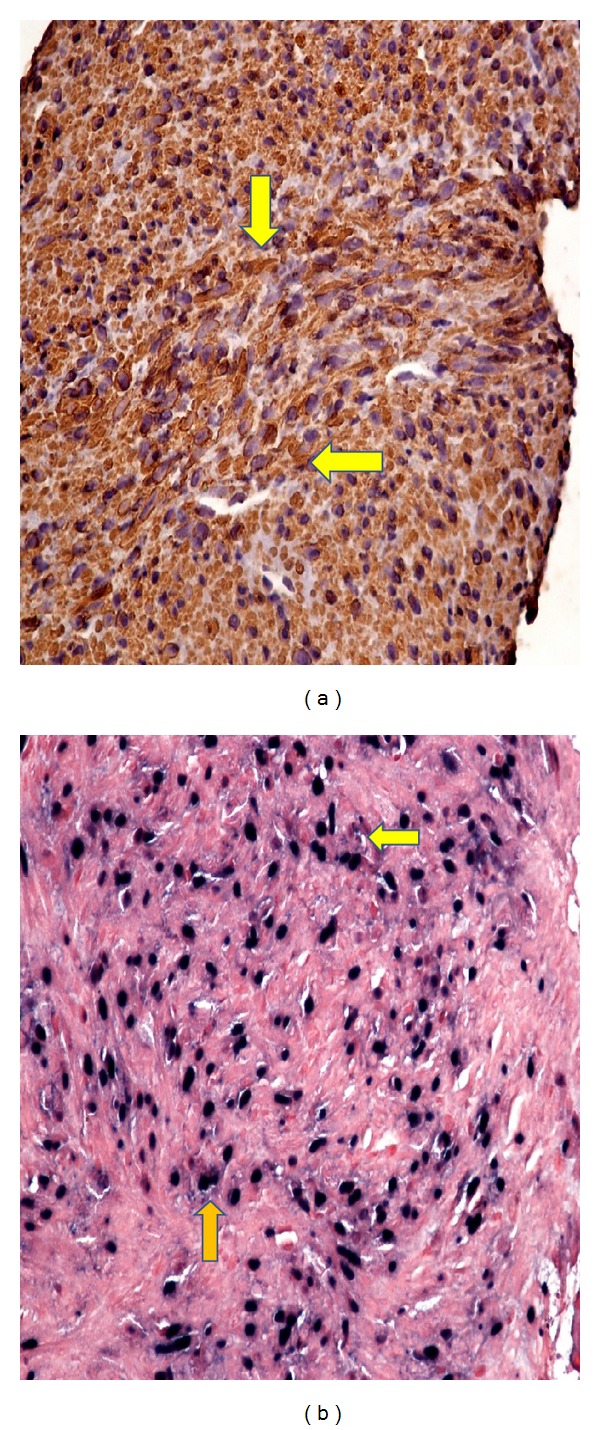
Immunohistochemical staining of the hepatic tumor. (a) Cytoplasm of the spindle shaped tumor cells stain positive for smooth muscle actin. (b) Neoplastic cells stain positive with nuclear in situ hybridization for EBV.

A 54- year- old African American man presented to our hospital with intractable hiccups that started about a week prior to admission. He also reported intermittent low grade fever with poor oral intake and weight loss of 9 kilograms for the last 3 months. He denied abdominal pain, nausea, vomiting, or irregular bowel habits. His comorbidities included HIV/AIDS, seizure disorder, and hypertension. His CD4 count was 24 at the time of admission. He had no history of liver disease or alcohol abuse. He never had any surgery. Family history was negative for any malignancies or liver disease. On physical examination he appeared cachectic. He had no palpable lymph nodes. Abdominal examination revealed minimal tenderness in the right upper quadrant and hepatomegaly of about 6 cm below the right costal margin. Laboratory analysis revealed alanine aminotransferase 81 IU/L (0–34 IU/L), aspartate aminotransferase 56 IU/L (0–40 IU/L), alkaline phosphatase 160 IU/L (28–94 IU/L), serum albumin 2.8 g/dL (3.4–5 g/dL). Serum bilirubin, prothrombin time, white blood cells, and platelets were normal. Septic workup including blood and urine cultures and chest radiograph were normal. Abdominal computed tomography (CT) of the chest and abdomen showed a hypodense rim enhancing lesion about 3.5 cm by 2.5 cm in size with a necrotic center within segment VII (Figure 1) and another 2.8 cm by 2.1 cm lesion in segment IVB of the liver. The lesion in segment IVB appeared to be nonspecific. He was initially treated with broad spectrum antibiotics for presumed pyogenic liver abscess. CT chest also revealed bilateral pulmonary nodules (Figure 2). Bronchoscopy revealed no endobronchial lesions and biopsies and lavage were negative for malignant cells. The nodules were deemed to be inflammatory changes. A CT guided biopsy of the liver lesion in segment VII revealed a tumor comprising spindle cells arranged in interlacing pattern (Figures 3(a) and 3(b)). There was minimal nuclear pleomorphism, tumor necrosis, and no increase in mitotic activity. Immunohistochemical stains were positive for smooth muscle actin (Figure 4(a)) but negative for desmin, CD117, and CD34. In situ hybridization for Epstein-Barr virus (EBV) encoded small nuclear RNAs (EBER) (Figure 4(b)). The neoplastic cells revealed strong nuclear stain consistent with evidence of EBV infection. These pathological features were consistent with a diagnosis of spindle cell neoplasm, low grade leiomyosarcoma involving the liver. Tumor markers, namely, alpha-fetoprotein, lactate dehydrogenase (LDH), and carcinoembryogenic antigen (CEA), were within normal range. Antibodies to hepatitis C and hepatitis B surface antigen were negative. He underwent esophagogastroduodenoscopy and colonoscopy which were unremarkable. Patient was offered further intervention with surgery but he refused and opted for conservative management. 

## 3. Discussion

Sarcomas constitute about 1-2% of malignant tumors of the liver [2, 3]. The most common type of primary malignant mesenchymal tumor is angiosarcoma (36%) followed by leiomyosarcoma (12%), fibrosarcoma (7%), and other sarcomas (44%) [4]. Most of the hepatic leiomyosarcomas are metastatic from gastrointestinal tract, genitourinary tract, uterus, retroperitoneum, and major blood vessels [1]. Extensive workup should be done with endoscopies, small bowel follow-through, abdominal, and chest imaging, and PET scan to rule out extra hepatic primary. Primary leiomyosarcoma of the liver arises from smooth muscle cells in the wall of intrahepatic blood vessels or biliary ducts [5, 6]. Potential etiological associations reported in the literature include EBV, HIV/AIDS, and history of immunosuppression.

Mean age of presentation as identified from various case reports is 58 years [7]. Hepatic leiomyosarcoma has also been reported in children in association with acquired immunodeficiency syndrome (AIDS) and Epstein bar virus (EBV). It is more common in the right lobe and found to be metastatic in about 40% at the time of initial diagnosis. Most often they are asymptomatic until they grow to a large size. No specific symptoms can be attributed as characteristic for this pathology. Patients usually present with variable symptoms of abdominal pain, bleeding, weight loss, and jaundice. Our patient presented with intractable hiccups. No specific tumor markers were identified that may help to diagnose primary leiomyosarcoma of the liver. LDH was identified to be elevated in more than 50% of patients. Liver function test might be deranged in few patients. The absence of any specific symptoms or serological markers makes the diagnosis of hepatic leiomyosarcoma challenging.

The findings of hepatic leiomyosarcoma on CT scan and MRI are non-specific. These tumors are found to be hypervascular on imaging. CT may reveal a large, well-defined, heterogeneous hypodense mass with internal and peripheral enhancement or cystic mass with an enhancing thick wall [8–10]. MR findings described are homogenous or heterogeneous hypointensity lesion on Tl-weighted images and hyperintensity on T2-weighted images with occasional observation of encapsulation [11, 12]. High index of suspicion and careful consideration of leiomyosarcoma as one of the differential in the evaluation of hepatic lesions may help in earlier diagnosis of these lesions.

Association of EBV infection and increased incidence of leiomyosarcoma in immunosuppressed patients such as HIV/AIDS and posttransplant is clearly documented and well researched in the literature. In situ hybridization studies of leiomyoma and leiomyosarcoma in HIV patients demonstrate EBV in the tumor cell, but not the adjoining normal cells. In AIDS patients the EBV receptor (CD21/C3d) may be upregulated allowing EBV to enter into muscles cells, contributing to malignant transformation. Similar pathogenesis of EBV induced leiomyosarcoma in iatrogenic immunosuppressed patients, especially in post-transplant patients, has been reported. There are isolated reports of decreased incidence of recurrence of leiomyosarcoma in EBV infected patients, in whom immunosuppression is decreased after transplantation. 

 If liver lesion appears malignant and deemed to be surgically resectable, there is no need for preoperative biopsy. In our case there was concern for abscess and hence CT guided aspiration was performed. Biopsy will reveal intersecting bundles of spindle cells with immunoreactivity to desmin, actin, and SMA but negative for keratin, S-100 protein, and neuron-specific enolase [13]. As in other soft tissue sarcomas, malignant potential is assessed by histological grading, size, and presence of mitoses. Hepatic leiomyosarcoma has four different histological types. Types 1, 2, 3, and 4 are defined as well differentiated, moderately differentiated, poorly differentiated, and myxoid leiomyosarcoma, respectively. Type 4 or myxoid leiomyosarcoma has an aggressive growth pattern [14, 15].

Therapeutic interventions include surgical resection, systemic chemotherapy, and liver transplantation. Best treatment modality is surgical resection with tumor-free margins which has good long term survival [16, 17]. In the evaluation of surgical treatment of primary hepatic sarcoma, only histological grading was found to be significant predictor of patient's survival [18]. In this same study, patient with high-grade tumor had 5 year survival rate of 18% and about 80% in low-grade tumors, following tumor-free resection [18].

Patterns of sites of recurrence of these tumors are similar to other sarcomas. Hepatic leiomyosarcoma tends to metastasize hematogenously to the lung, but lymphatic metastasis and peritoneal seeding have been described. Adjuvant chemoradiotherapy had been recommended for eradication of microscopic residual disease. However the success of this therapy in achieving local control and prolonged patient survival needs to be further studied although a meta-analysis has shown a small benefit [19]. Postoperative chemoradiotherapy has been tried without much success. Chemoembolization of metastatic hepatic sarcomas has been shown to increase the survival [20]. Primary hepatic leiomyosarcoma is known to be a hypervascular tumor and this approach needs to be evaluated for further management of nonresectable lesions.

The role of liver transplantation remains controversial. In the study by Husted et al., six patients with primary hepatic angiosarcoma and thirteen patients with metastatic sarcoma underwent liver transplantation and the 1-, 3-, and 5-year survival was 47%, 15%, and 5%, respectively. Recurrence rate was 95% after a median interval of 6 months. Given the high tumor recurrence rate and poor survival, Husted et al. discouraged liver transplantation for the management of primary or metastatic sarcoma of the liver [21]. However reducing the level of immunosuppression improved patient survival despite tumor recurrence and this was reported by Rajput and Kraybill [22] and supported by Brichard et al. [23].

## 4. Conclusion

High index of suspicion and prompt investigation will help to diagnose primary hepatic leiomyosarcoma at an earlier stage. Surgical resection without any residual tumor has clearly been demonstrated for prolonging patient's survival. Use of neoadjuvant, adjuvant chemoradiotherapy, and hepatic artery chemoembolization needs to be further evaluated.
